# Burnout Syndrome in Sport-Related Jobs: A Meta-analysis of Recent Evidence

**DOI:** 10.1007/s40279-026-02463-y

**Published:** 2026-06-04

**Authors:** Valentina Viego, Juliana Aparecida de Oliveira Camilo, Braz Isac Andrade Santos, Ícaro Lima-Sena, Carolina Hamodi

**Affiliations:** 1 Department of Economics, Instituto de Investigaciones Economicas y Sociales del Sur, IIESS, Universidad Nacional del Sur, San Andres 800, Campus Universitario Palihue, 8000 Bahia Blanca, Argentina; 2 Universidad de Valladolid. Business and Work Sciences, Campus Duques de Soria, 42004 Soria, Spain; 3 Postgraduate Program in Psychology, Universidade Federal da Bahia, R. Pedro Vieira Borges, Salvador, Brasil; 4 Department of Sociology and Social Work, Universidad de Valladolid, Campus Duques de Soria, 42004 Soria, Spain

## Abstract

**Background:**

Burnout syndrome in sports is receiving increasing attention in the empirical literature applying psychometric tools. Since 2019, the number of scientific publications has doubled. This growth has increasingly dissociated athletes from their status as workers and burnout is rarely conceived as a phenomenon emerging from a working relationship. This study aims to meta-analyze the empirical measurements of burnout using scales in athletes and occupations related to professional sports during 2014–2023.

**Results:**

The initial search detected 996 studies. After a screening guided by PRISMA principles, we meta-analyzed 113 independent studies comprising 133 burnout measurements from 35,059 athletes, coaches, and referees across 29 countries. The results show a generalized use of the Athlete Burnout Questionnaire (ABQ), a notable heterogeneity in the estimates, signs of publication biases in some specific subscales, higher mean scores in personal accomplishment than other burnout dimensions, a decreasing trend in global scores over time, a higher burnout prevalence in developed countries, and different mean scores according to the scale applied.

**Conclusions:**

The results highlight the need to continue improving the existing psychometric tools and focus interventions on the perception of accomplishment to reduce burnout incidence.

**Supplementary Information:**

The online version contains supplementary material available at https://doi.org/10.1007/s40279-026-02463-y.

## Key Points

**Table:** 

Overall burnout levels among professional athletes tend to be low to moderate, with some evidence of a declining trend in recent years.
Age and socioeconomic context emerge as the main moderators of burnout, as younger athletes and those in developed settings report higher levels of exhaustion.
There are signs of publication bias—especially for exhaustion and global burnout scores—suggesting that small studies may overestimate the true magnitude of burnout in sport.

## Introduction

Burnout syndrome (BS) is recognized by the World Health Organization as a work-related condition and was incorporated into the ICD-11 classification in 2022 (code QD85) [1]. It is characterized by emotional exhaustion, detachment, and reduced personal accomplishment, emerging from chronic tension between individuals and their work environments [2–4]. Originally described in occupational contexts, burnout has progressively been examined across a wider range of high-demand activities involving sustained emotional, cognitive, or physical effort [5, 6].

In recent decades, research has increasingly addressed burnout in the sports domain, where prolonged performance pressure, competitive stress, and chronic training demands create conditions conducive to psychological exhaustion [7, 8]. Studying burnout in athletes is essential for understanding mechanisms that undermine well-being, performance sustainability, and long-term participation, as well as for informing prevention strategies and institutional interventions.

This study focuses on professional athletes, understood as workers whose activity is contractually regulated and involves labor demands comparable to other high-intensity occupations. We aim to meta-analyze the prevalence of BS and its associated factors in this population, addressing the following research questions:

- What psychometric instruments are used to measure burnout in professional athletes, and do systematic differences emerge among them?
- What is the prevalence of burnout in professional athletes, and are there potential biases in the studies reporting these estimates?
- How do factors such as year of publication, age, sex, type of sport, and socioeconomic context influence burnout levels?

Despite the growing literature on burnout in sports, two important limitations persist. First, most existing reviews and meta-analyses rely on pre-pandemic data, overlooking the potential impact of COVID-19 on athletes’ mental health and occupational conditions. Second, previous meta-analyses have not systematically assessed publication bias, which may distort pooled prevalence estimates. This study addresses these gaps by updating the temporal coverage to include the post-pandemic period and by applying appropriate statistical tools to examine publication bias and moderators of burnout prevalence.

## Conceptual Issues

After the introduction of the notion of burnout by Freudenberger and Maslach [1, 2], it was proposed to decompose the syndrome into three components: emotional exhaustion, depersonalization, and reduced personal accomplishment. Emotional exhaustion refers to the feeling of being emotionally drained and overwhelmed (e.g. extreme fatigue), which can lead to a decrease in the ability to empathize and care for others. Depersonalization manifests in negative, cynical, and/or distant attitudes (e.g. loss of interest) toward colleagues or clients. Finally, reduced personal accomplishment relates to the feeling of a lack of achievement and self-devaluation at work.

Based on these dimensions, Maslach and Jackson [9] proposed the index known as the "Maslach Burnout Inventory" (MBI), with three subscales capturing these dimensions of BS (emotional exhaustion, depersonalization and reduced sense of accomplishment). This inventory has become one of the most widely used tools for measuring burnout syndrome [8, 10], and has contributed to the development of several other quantitative instruments and inventories. The MBI consists of 22 items referring to feelings and thoughts related to work, which are assessed using a 6-point scale ranging from 'never' to 'daily'. The items are grouped into the three subscales already referred to. Scores are calculated by summing the items for each subscale, with higher scores indicating greater levels of burnout. Although the MBI is considered the gold standard, it is insufficient for diagnosing BS. Various studies highlight the limitations of the MBI index as an instrument to measure BS, which has fostered the development of other instruments to measure and diagnose the BS [11]. First, the MBI was conceptualized and validated within traditional occupational contexts, which raises doubts about its capacity to capture sport-specific stressors related to performance pressure, competition cycles, or training load. Studies from elite sports programs in Spain and New Zealand [11, 12], among others, have shown that key dimensions of the MBI—particularly depersonalization—do not align well with how athletes experience emotional distancing or disengagement from sport. A second line of criticism concerns cultural and linguistic variability: cross-cultural validations conducted in Asia and Latin America suggest that certain MBI items may not adequately reflect culturally embedded norms of emotional expression, collective responsibility, or coach–athlete relationships [13]. This has resulted in mixed evidence regarding measurement invariance across regions, sporting disciplines, and competitive levels and led to development of specific tools to measure BS in sports.

Kristensen et al. [15] highlighted that MBI focuses mainly on workers in education or health services but does not adequately capture the chronic fatigue experienced by workers outside those industries. For this reason, they proposed an alternative scale known as the Copenhagen Burnout Inventory (CBI). The CBI was developed as a more comprehensive tool to measure BS and includes three subscales: personal burnout**,** work-related burnout, and client-related burnout. The personal burnout subscale assesses the level of exhaustion a person feels regardless of his/her work context, making it applicable to any individual. Work-related burnout, on the other hand, focuses on exhaustion directly linked to professional responsibilities, while the client-related burnout subscale measures emotional fatigue associated with interactions with clients, patients, students, or other groups frequently engaged with the worker. The three CBI subscales have demonstrated high internal reliability and the ability to predict health problems, such as sickness absences and sleep disorders.

The review offered by Shirom [16] states that the MBI focuses mainly on emotional exhaustion and underestimates physical or cognitive exhaustion. Thus, the BS may be underestimated in populations where these types of exhaustion are more prominent (manual work and/or jobs with less emotional implication). This critique has led to the proposal by Shirom and Melamed [17] known as the Shirom-Melamed Burnout Measure (SMBM). This scale is based on the theory of Conservation of Resources, which emphasizes the depletion of an individual’s energetic resources due to chronic stress. Different from the MBI, the SMBM measures physical fatigue, emotional exhaustion, and cognitive weariness. This instrument contains 14 items distributed across these three subscales and is answered on a 7-point Likert scale (1 = Never; 7 = Always). The SMBM has demonstrated strong psychometric properties, including reliability and construct validity, and is particularly suitable for occupations with high physical and cognitive demands, making it an effective alternative to the MBI in diverse professional settings [17].

Athletes, like other professionals, experience burnout but face specific factors such as external pressure from coaches and families, perfectionism, and an external locus of control. By the early 1990s, Dale and Weinberg [18] provided a comprehensive review of BS in the sports field, pointing out that it emerges as a reaction to chronic stress characterized by physical, mental, and emotional exhaustion. The authors explore how the interaction between personal and situational factors contributes to BS in athletes, coaches, and sport-related personnel. They also address individual differences, including gender, in the perception of BS, and emphasize the lack of consensus on its operational definition. During the 1980s and 1990s, the MBI was the most used tool to assess BS. Thus, lack of a specific instrument for athletes revealed a research gap [19].

Raedeke [20] recognizes that physical fatigue resulting from training and competition increases the domain of the emotional exhaustion dimension proposed by Maslach and Jackson. Thus, Raedeke and Smith [21] proposed the Athlete Burnout Questionnaire (ABQ), another widely used instrument to measure BS in the sports context. It consists of 15 items organized into three factors: reduced sense of accomplishment, physical and emotional exhaustion, and devaluation of sports practice. They adjusted the personal accomplishment dimension of the MBI to reflect feelings of inefficacy regarding performance and athletic achievements. In the ABQ scale, that factor is known as “reduced sense of accomplishment”. Finally, the most significant change introduced by ABQ is related to the depersonalization dimension, which was replaced by the notion of devaluation of the practiced sport. While depersonalization is considered relevant in the context of human services, Raedeke and Smith suggest that it is not as pertinent in the sports domain [22].

Another instrument commonly used in the sports context is the Burnout Inventory for Athletes (IBD), suggested by Garcés de Los Fayos [23]. It consists of 26 items designed to measure the same three dimensions of BS proposed by Maslach and Jackson [9]. This instrument was later revised as the Revised Burnout Inventory for Athletes (IBD-R, see [24]). The revised version reduced the number of items to 19, making it easier to administer and decreasing the time required for completion.

The Sport Burnout Inventory Dual Career (SpBI-DC) was proposed by Sorkkila et al. [25], acknowledging that most athletes are young and often navigate a dual career as students and professional athletes. The authors argue that part of the BS originates from the need to balance education commitment with training and performance in sports. Traditional instruments would not sufficiently cover this aspect; thus, the authors suggest combining psychometric tools to measure BS in education and sports contexts. The authors propose a battery of 10 items with three subscales like those included in the MBI or ABQ.

In sports, coaches and trainers have received more attention than athletes concerning BS. Thus, inspired by ABQ, the specific quantitative tool to measure BS in athletes, other studies have proposed analogous tools for related occupations in sports environments, like referees or coaches. In fact, Harris and Ostrow [26] proposed the Coach Burnout Questionnaire (CBQ), which is an adaptation of the ABQ to the context of coaches. The CBQ reflects the experience of coaching rather than practicing the sport. The authors report that the instrument was reviewed by a panel of experts, who verified the clarity and adequacy of the adaptation, ensuring its content validity. The CBQ consists of 15 items, organized into three traditional dimensions; exhaustion, reduced sense of accomplishment, and devaluation. Each dimension contains five items. The items of the CBQ are answered on a 5-point Likert scale, ranging from "Almost never" to "Almost always". The CBQ has content validity and is widely used to assess emotional and professional BS in sports coaches, although studies like that of Yan et al. [27] suggest the need for further validation in specific cultural contexts, such as the Chinese.

Likewise, the so-called Burnout Inventory for Referees (BIR) was developed by Brandão et al. [28] to assess BS specifically in soccer referees with the three dimensions proposed in the MBI. The replicable structure of the BIR was assessed by confirmatory factor analysis. In addition, the authors illustrated that the total BS score was positively correlated with negative mood states (e.g. tension, depression, anger, fatigue, and confusion) and negatively correlated with vigor. The instrument was developed in Portuguese and was adapted to the Brazilian context, with semantic and conceptual equivalence.

Empirical literature on BS in athletes has increased significantly from the 1980s to the present. Goodger et al. [29] included 58 studies about BS in sports published from 1989 to 2005, including in athletes (*n* = 27), coaches (*n* = 23), sports directors (*n* = 2), fitness trainers (*n* = 2), referees (*n* = 3), and sports center employees (*n* = 1). The analysis highlighted that most research utilized self-report measures and cross-sectional designs. The main factors associated with burnout were categorized as psychological, demographic, and situational, with varied results (positive, negative, inconclusive, and unrelated). The authors concluded that, despite the growing interest in the subject, significant gaps remain, particularly regarding longitudinal and qualitative research, as well as specific interventions for the prevention and treatment of BS in sports. Also, they state that the empirical basis for BS is still small, comparing more than 6000 publications on the topic with less than 100 empirical studies.

García-Parra et al. [30] note that in the past three decades, the study of BS in the sports field has experienced significant growth, encompassing athletes, coaches, managers, and referees. In fact, they identified 248 articles published between 1981 and 2015 and analyzed 104 of these in depth. One of the main findings was the inconsistency between the results of the studies; some indicate that BS is more prevalent in women, while others did not find significant gender differences. Similarly, some studies point to a correlation between training load and BS, while others did not find such an association. The authors also point out a tendency to examine existing models rather than propose new ones. Stress and motivation variables remain central to understanding BS, with growing interest in theories of goal achievement and self-determination. Finally, regarding assessment, the authors state that there are sufficient instruments, suggesting the exploration of new theoretical models, going further into the study of personality variables, and developing and validating prevention and intervention programs—an area currently showing a lack of empirical studies.

Meta-analysis about BS is more recent. Madigan et al. [31] identified 91 studies and 396 effect sizes for more than 21,000 athletes reported during 1997–2019, in a leading meta-analysis. One of their main findings was an increasing trend in BS scores, particularly in the reduced sense of achievement and devaluation subscales. However, levels of physical and emotional exhaustion, as well as overall burnout, did not show a time trend.

Similarly, Woods et al. [32] analyzed 59 studies but focused exclusively on those using the ABQ in team sports following PRISMA guidelines. The authors synthesized 123 variables potentially correlated with BS, and 18 were found significant. In particular, they found negative associations between burnout and autonomy, competence, self-determined motivation, and social support and positive associations with (de)motivation, socially prescribed perfectionism, and a controlling coach style. The narrative synthesis addressed the remaining variables. The review emphasizes the need for replication and greater representation of female athletes.

Another meta-analysis related to BS is that of Glandorf et al. [33]. However, this review does not focus on BS measures but rather on its effects. After analyzing 56 studies, they found evidence that BS negatively influences mental health, leading to depression, anxiety, and physical symptoms. Nevertheless, the heterogeneity of effects was considerable, highlighting the need for further research with more rigorous designs to better understand the triggers and consequences of BS.

Carlin and Garcés de los Fayos [34] analyzed the historical evolution of BS, from its initial identification in labor contexts to its application in sports during the 1980s. They found that athletes suffer similarly from BS, which frequently leads to diminished performance and early dropout from sports. Over time, theoretical models have been proposed to explain the syndrome in athletes, including factors such as overtraining, competitive stress, and social relations, pointing out that labor burnout and sport burnout share similar aspects. In both environments, the search for perfection and constant effort foster conditions for the emergence of BS. Nevertheless, there is criticism that recent studies in the sports field have overlooked the concepts of ‘work’ and ‘worker,’ which were essential in the original research on BS, even though professional athletes face challenges like those of a worker in terms of effort and commitment. This omission could limit a comprehensive understanding of the syndrome in this context.

The neglected ‘work–sport’ perspective in research on athlete burnout stems from the historical tendency to conceptualize athletes’ exhaustion within sport-specific psychological frameworks rather than through occupational health models. As a result, athletes have often been treated as a special case, separate from the broader workforce, despite the fact that their training, performance pressures, and employment-related demands exhibit clear parallels with work-related stress processes. Moreover, the development of the sports sciences and occupational health literature has occurred mostly in parallel, with limited cross-fertilization. Consequently, the potential of the work–sport approach—linking athletes’ burnout to employment conditions, job insecurity, workload, or organizational environment—has not been fully explored, even in the post-recognition era following the WHO’s classification.

## Materials and Methods

### Search Strategy

We searched databases recognized for their coverage and prestige in the academic field—BVS, Embase, Medline/PubMed, PsycInfo, Sage Open, Scopus, and Web of Science. The choice of these platforms aimed to ensure methodological quality and the inclusion of a representative diversity of perspectives and contributions on burnout in athletes. These databases are distinguished by their extensive multidisciplinary coverage, rigor in peer-reviewing, and access to vast academic literature. Additionally, they offer global coverage, allowing the inclusion of studies conducted in different geographical and cultural contexts. They also enable a comprehensive search for studies addressing athlete burnout from various perspectives, including health, psychology, sports, and other relevant disciplines.

To optimize the search, descriptors were formulated in three different languages: English, Spanish, and Portuguese. The keywords were combined using the Boolean operator AND, forming the following descriptor arrays: ‘Burnout Syndrome’ AND ‘Athletes’, ‘Síndrome de Burnout’ AND ‘Deportistas’, ‘Burnout’ AND ‘Atletas’. This strategy was implemented to capture the specific nuances of the literature in different languages.

The search period covered publication dates between January 2014 and December 2023. The study was conducted following the PRISMA guidelines (Preferred Reporting Items for Systematic Reviews and Meta-Analyses) proposed for systematic reviews [35].

### Eligibility Criteria

We included studies published in English, Spanish, or Portuguese. Participants should be professional athletes, coaches, or referees. Although in this study we occasionally refer to sport-related occupations, we did not identify—nor therefore include—studies that measure BS in administrative positions, medical support staff, managers, event logistics personnel, or sport service roles. We understand that BS is more likely to affect athletes, coaches, and referees than other occupations within the sport sector.

We considered professional athletes as those who maintain an employment relationship with a sports organization, governed by specific regulations. This relationship is characterized by voluntariness, regularity in practices and competitions, the athlete's dependence on the organization, and monetary compensation [36]. The relationship with a sports organization is not exclusive of professional status, particularly in amateur competitions that offer prizes in events. This latter characteristic is common in combat sports, adventure sports, beach sports, or dance. These athletes, often marginalized, may face precarious training conditions and/or issues related to illegal substances [37]. Thus, if the athlete does not hold a formal contract with a club or league, his/her professional character should be demonstrated by competing in recognized professional events requiring official accreditation. Finally, the third criterion for accomplishing professional status is receiving monetary income from sport. Professional athletes are not limited to elite sports, as their personal or family income may depend on competitions not considered top-tier. Ultimately, we define professional athletes as those who engage in competitive sports as a livelihood, without recreational, educational, or health-related purposes.

We excluded studies a) published in books (parts or complete), reviews, dissertations, and theses; b) that included sports activities carried out in non-professional or competitive spheres; and c) that used secondary data.

### Data Extraction and Quality Assessment

**Fig. 1:**
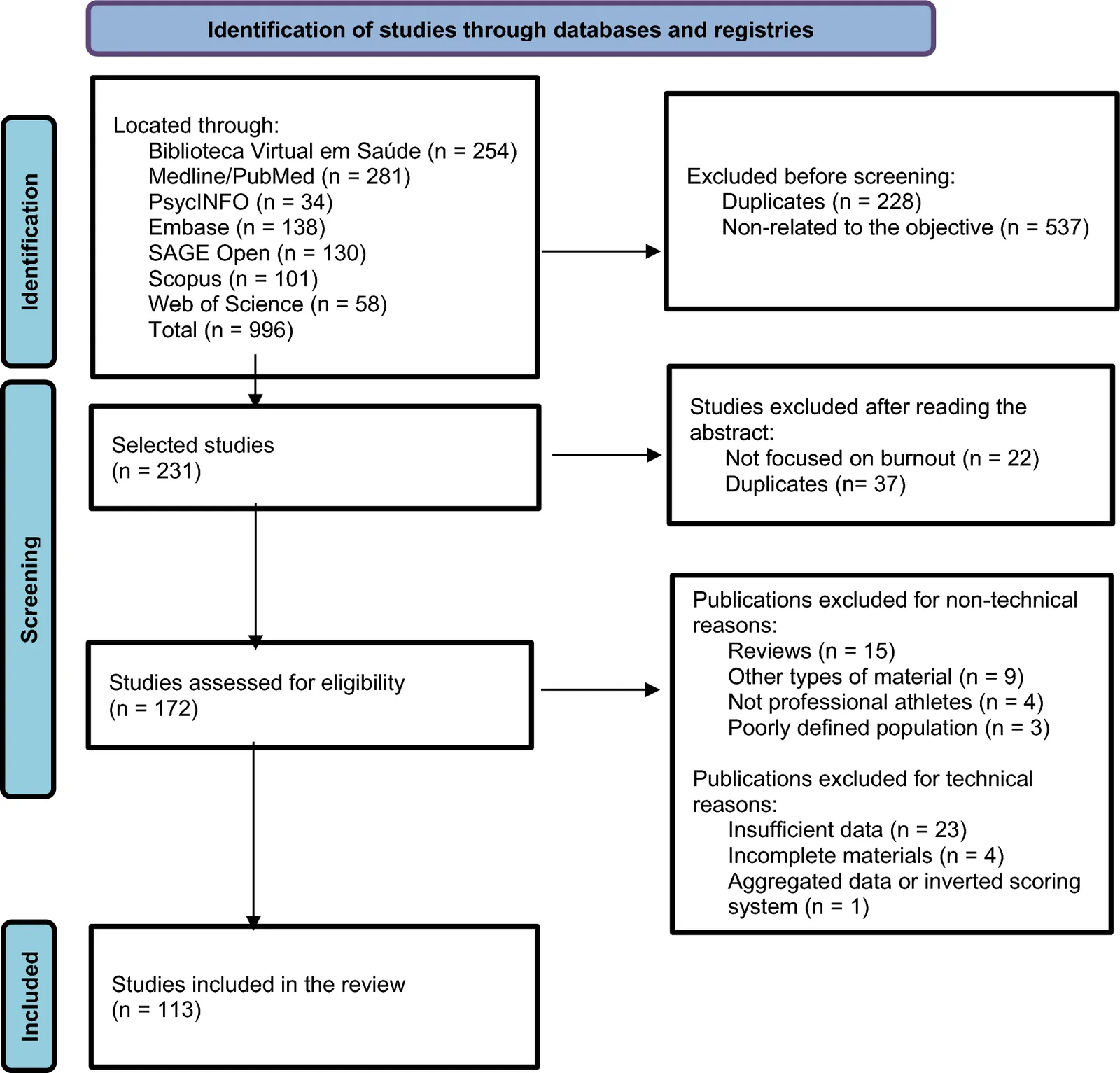
Flow chart for identification, screening, and eligibility of studies according to PRISMA recommendations

The initial search detected 996 studies (see Fig. 1) selected based on title or abstract. During the screening stage, a criterion for exclusion was lack of relevance to the review topic. Many studies do not directly address burnout in athletes (i.e., they investigated other populations such as healthcare or education professionals). Other studies focused on different aspects of mental health in athletes, but not specifically on burnout. The organization and analysis of these results were recorded using Excel software, providing a detailed view at the end of the search process.

Before the screening, we also excluded studies with the same title. The screening advanced to the reading of abstracts, during which we found another 37 duplicates not detected during the identification phase because of language variations. In addition, we excluded 22 studies not focused directly on BS.

In the next phase, we excluded 15 studies classified as documentary and review materials and nine reports that were not original research articles. We also excluded seven studies after a careful reading and finding that athletes were not professional or the population was not clearly defined.

In the final stage of article selection for the research, rigorous criteria were applied to ensure that only quantitative studies with sound methodology were included in the meta-analysis. This phase was crucial to guarantee the quality and relevance of the data used in the analysis. During this stage, 28 studies were excluded for technical reasons and lacking data (did not report estimations and/or standard deviations) or materials (dimensions within scales, number of items included in the scale, range of responses, etc.) and deficient treatment of data.

Firstly, articles addressing amateur or non-professional athletes were excluded, as the research was focused exclusively on professional athletes. This profile was essential to ensure the relevance of the data and the accuracy of the analysis, as factors affecting burnout and mental health can vary significantly between professional and amateur athletes.

Additionally, one article was discarded due to inconsistent data presentation, specifically an inverted ordering of items related to burnout. In this study, lower values indicated higher burnout, which caused confusion in interpreting the results and compromised the accuracy of subsequent analyses. Errors of this type in data presentation can severely affect the validity of the results, making the inclusion of this article inappropriate.

Another reason for exclusion was inadequate or poorly defined description of the studied population. Some articles did not provide clear information on whether the participants were professional athletes or sports students, making it difficult to compare and analyze the data. The lack of clarity regarding the participants’ profiles can lead to misinterpretations and compromise the analysis focused solely on professional athletes.

Finally, a significant portion of the articles was excluded due to insufficient data for a complete analysis. Many studies did not present essential statistical measures, such as means and standard deviations, or did not provide the full data necessary for evaluation. Moreover, difficulties in accessing the required information contributed to excluding these articles. The absence of robust and complete data limits the ability to conduct a thorough analysis. Therefore, ensuring that the final analysis was based on substantial and reliable data was fundamental to the quality of the research.

Some studies reported various measures of BS for different moments in repeated samples (i.e. rest, training, tournament, post-tournament). In those cases, we chose the measurement taken during training, outside of competition periods, to avoid the influence of competitive results for the athletes at that moment on BS scores. Likewise, we did not consider BS measurement during vacation periods.

The identification and screening were conducted by two independent members of the research team. At the end of each stage, the team maintained discussions to report the results and warrant the reliability and robustness of the procedure.

We considered a wide range of burnout scales. As previously mentioned, the most popular burnout scale is the ABQ, which contains 15 items grouped into three dimensions: reduced sense of accomplishment (RSA), sport devaluation (DEV), and emotional/physical exhaustion (EPE). Another known burnout scale is the IBD-R, composed of 19 items in three subscales: professional efficacy, depersonalization, and emotional exhaustion. These subscales are also included in the MBI tool, which usually applies 22 items. When applied to sport, these subscales can be comparable to the ones considered in the ABQ measure. In the few cases where the scales did not offer a structure based on those three comparable subscales, we considered their global scores.

It should be noted that while higher scores in DEV and EPE subscales indicate higher stress levels, the subscale RSA has a decreasing order, whereby higher marks denote lower BS levels and vice versa.

Usually, replies from participants are registered on a 5-point Likert scale. If the final scores are computed as averages, their range is 1–5. Other studies simply add the scores from each item in each dimension. Thus, the range of the global scores can be 15–75 or 19–95. If the studies applied a 7-point Likert scale, ranges may vary from 7–105 (averages with 15 items) to 19–133 (sums with 19 items). Also, some studies exhibit their scores on a 0–100 scale. Following standard practice in psychometric meta-analyses [38], we transformed the measures into a 5-point scale centered on 0 within a range (− 2, 2) in order to get comparable measures of BS. This procedure eliminates scale-driven variance and ensures that cross-study differences reflect substantive rather than purely metric discrepancies. A centered scale (− 2 to 2) further facilitates the interpretation of pooled effects, as 0 represents the midpoint and negative/positive values indicate lower/higher burnout relative to this common reference point.

The formula to re-express the scores is

$${\mathrm{BS}}_{{\mathrm{n}}} = - {2} + \left( {{4}/{\mathrm{range}}_{{\mathrm{o}}} } \right)*\left( {{\mathrm{BS}}_{{\mathrm{o}}} - {\mathrm{min}}_{{\mathrm{o}}} } \right)$$

The standard deviation was re-expressed as

$${\mathrm{BD}}_{{\mathrm{n}}} = \, \left( {{4}/{\mathrm{range}}_{{\mathrm{o}}} } \right)*{\mathrm{BD}}_{{\mathrm{o}}}$$

where BS = burnout mean score, BD = burnout standard deviation, n = subscript for new measure, o = subscript for original measure.

It is important to mark that the purpose of the (− 2, + 2) transformation is not to impose diagnostic equivalence across instruments, but rather to enable operational comparability for meta-analytic synthesis, allowing effect sizes to be analyzed on a common metric. This harmonization is purely statistical and does not imply conceptual or clinical equivalence between measurement tools. As rescaling cannot eliminate intrinsic distributional differences arising from instrument-specific properties (e.g., item wording, response formats, scale ranges, sensitivity, or ceiling/floor effects), the instrument type is explicitly accounted for in the analytical framework with different coding, thereby modelling measurement heterogeneity rather than assuming full equivalence. In this sense, the harmonization procedure serves as a pragmatic methodological strategy for synthesis, while measurement non-equivalence is treated as a source of structured heterogeneity rather than ignored. The interpretation of results is therefore framed at the level of the general burnout construct, not at the level of diagnostic or clinical comparability across instruments.

We conducted a meta-analysis to assess the magnitude and heterogeneity of burnout among athletes. The size of burnout is computed as

$$\widehat{B}=\frac{{\sum }{w}_{i}{\mathrm{B}\mathrm{S}}_{i}}{{\sum }{w}_{i}},$$

where BS_*i*_ is the burnout measure emerging from each study and it is weighed by the inverse of its variance, *w*_*i*_. In turn, each *w*_*i*_ is computed as

$${w}_{i}=\frac{1}{{\sigma}_{i}^{2}+{\tau }^{2}},$$

where ${\tau }^{2}$ denotes the variability between studies.

We computed ${\tau }^{2}$ through the Restricted Maximum Likelihood estimator (REML), the statistic *I*^2^, which expresses the proportion of variability between studies, and the Cochrane’s *Q* test which tests if observed variability in studies is higher than expected by chance.

Most studies reviewed published one estimate of burnout but some of them report two or three different estimations for different subsamples. In these cases, we stored the results with the same study ID code to admit dependence between results.

We assessed the bias risk or small-study effect through funnel plots. To explain variability across results we conducted a multilevel meta-regression. The multilevel nature emerges from the fact that some studies provide more than one estimate of burnout, so their results are not independent. The equation that expresses the multilevel nature of the meta-regression is as follows:

$$\widehat{{\mathrm{B}\mathrm{S}}_{jk}}=\mathrm{B}\mathrm{S}+{u}_{j}^{3}+{u}_{jk}^{2}+{\varepsilon}_{ij}$$

where $\widehat{{\mathrm{B}\mathrm{S}}_{jk}}$ is the burnout score from the estimation k from the *j* study. There are two levels of variability, one emerging from the estimation *k* and another from the study. Also, ${\varepsilon}_{ij}$ denotes a classical error term with *N*(0, *σ*^2^). In meta-regression schemes, theta is explained with moderators.

We considered the following moderators for heterogeneity: year of publication, sample size, type of instrument used, average age of athletes, proportion of women included in the study, the type of activity (referee, coach, individual games, group games, multi-sport), if the study was conducted in a developed country, and if it conceives the athletes as workers and/or burnout as emerging from a work relationship between athletes and their employers (clubs, sponsors, etc.).

Also, we have checked the publication bias through funnel plots and the Egger test. It must be recognized that there are no versions of Egger tests available for dependent samples like the ones we have for studies with multiple estimations. That assessment was carried out with classical tools. Estimations were carried out with the package metafor in R.

## Results

We included 113 studies, giving rise to 133 burnout scale estimates comprising 35,059 professional athletes. As the number of studies is large, we do not exhibit the forest plot. Studies were conducted in 29 different countries, mainly from developed regions.

**Fig. 2:**
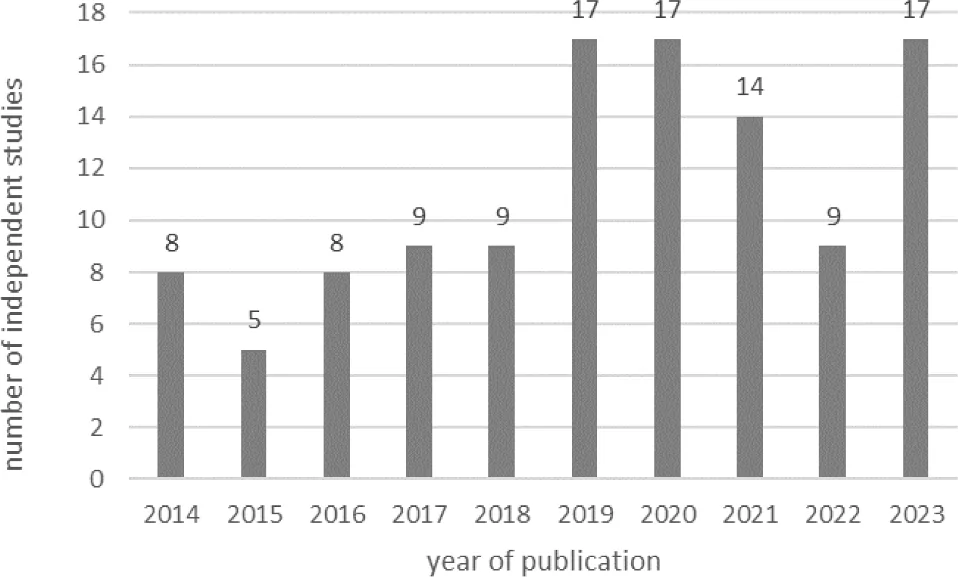
Number of independent studies measuring burnout syndrome in professional athletes by year of publication

The number of independent studies grew sharply in 2019 from a rather stable average of nine studies per year to 17. In particular, 2020 experienced a peak of 20 observations from 17 independent studies (Fig. 2). The significant increase in the number of studies since 2019 reflects a growing interest and recognition of the importance of burnout in the sports field research.

Most studies were published in English (81%), followed by studies in Spanish (~ 9.7%) and Portuguese (~ 8.9%). The most commonly used instrument to measure burnout in the studies is the ABQ, employed in 76.7% of cases. The IBD-R and the MBI were applied in 8.3% and ~ 6% of cases, respectively. Additionally, nearly 9% of the studies utilized other instruments. The average study sample size is ~ 284 participants, ranging from 8 to 2612. The average age of participants is ~ 20 years, ranging from 13.5 to 42.9 years old. On average, women represented around 38.5% of participants, with studies ranging from 0 to 100% of female participants. The studies focused on various types of sports activities. The majority evaluated multiple sports (43.9%), followed by individual sports (25.4%) and team sports (21.5%). A small percentage of studies focused on referees (1.5%) and coaches. We grouped the categories of referees and coaches to avoid micronumerosity issues. Seventy-five percent of the studies were conducted in developed countries. Less than 9% of the studies considered the professionals as workers, specifically relating burnout to their working relationship with employers (Table 1).

**Table 1:** Descriptive statistics of the meta-analyzed studies

	Valid cases	%/mean	Min	Max
Psychometric instruments used to measure burnout in professional athletes (Research question 1)				
Instrument applied				
ABQ	102	0.767	0	1
IBD-R	11	0.083	0	1
MBI	8	0.060	0	1
Other	12	0.090	0	1
Sample size, mean	133	283.7	8	2612
Work-related^a^	113	0.089	0	1
RSA, mean score	92	0.038	− 1.669	1.83
DEV, mean score	91	− 0.492	− 1.961	1.85
EPE, mean score	95	− 0.270	− 1.843	1.93
BS prevalence in professional athletes (Research question 2)				
Global burnout score	85	− 0.208	− 1.453	1.83
Factors that potentially influence burnout levels (Research question 3)				
Year of publication, mean	113	2019	2014	2023
Language^a^				
English	92	0.814	0	1
Portuguese	10	0.089	0	1
Spanish	11	0.097	0	1
Age of participants, mean	111	20.1	13.5	42.9
Proportion of women, mean	122	38.5	0	100.0
Type of activity assessed				
Referee	2	0.015	0	1
Coach	10	0.077	0	1
Individual sports	33	0.254	0	1
Team sports	28	0.215	0	1
Multi-sports	57	0.439	0	1
Developed countries^a^	110	75.45	0	1

*ABQ* Athlete Burnout Questionnaire, *BS* burnout syndrome, *DEV* depersonalization/devaluation, *EPE* emotional and physical exhaustion, *IBD-R* Revised Burnout Inventory for Athletes, *MBI* Maslach Burnout Inventory, *RSA* reduced sense of accomplishment

^a^Computed for independent studies, excluding repeated measures from the same study

Most studies report mean values for each of the three subscales commonly included in burnout indices. BS scores are reported across three different dimensions; for the one linked to an RSA, the mean score is ~ 0.04 indicating low levels of burnout in this dimension. The average score for DEV is − 0.49. The mean score related to EPE is − 0.27. Relatively fewer studies report the overall score of the BS scale, except perhaps when burnout is included as an explanatory in a regression model and/or is considered dependent on or influenced by other variables. In those cases, BS is often considered globally, without distinguishing its dimensions, and its average is near − 0.21. As the range of variation was centered on the 0 (moderate BS), the negative mean value for DEV, EPE, and global BS indicates that studies tend to find relatively low figures of burnout in those dimensions. Still, while overall estimates of BS, DEV, and EPE subscales depart significantly from 0 (*p* < 0.001) with negative mean values suggesting that burnout is in general low, the mean scores for RSA do not (*p* = 0.63), suggesting that RSA faces moderate levels of burnout, which is higher than that observed for the rest of the dimensions.

Table 2 exhibits the basic indicators for meta-analysis. As meta-analysis estimates are weighed by standard deviation, averages are not equal to the ones reported in Table 1. Thus, the ordering of the different dimensions of BS are slightly different from the ones exhibited in Table 1; DEV scores do not differ substantially from the EPE figures.

**Table 2:** Meta-analysis results

	RSA	DEV^a^	EPE^a^	Global
Score (95% CI)	− 0.026 (− 0.214 to 0.163)	− 0.57 (− 0.759 to − 0.381)	− 0.377 (− 0.560 to − 0.195)	− 0.029 (− 0.434 to − 0.146)
Estimated amount of total heterogeneity, tau^2^ (SE)	0.425 (0.12)	0.295 (0.12)	0.296 (0.12)	0.192 (0.0632)
Total heterogeneity/total variability, I^2^, %	72.05%	41.28%	42.37%	74.44%
Relative heterogeneity measure, H2	3.58	1.70	1.74	
Q test (*p*-value)	563.52 (< 0.0001)	151.576 (< 0.0001)	161.368 (< 0.0001)	571.577 (< 0.0001)
k	91	90	94	84

*CI* confidence interval, *DEV* depersonalization/devaluation, *EPE* emotional/physical exhaustion, *REML* restricted maximum likelihood, *RSA* reduced sense of accomplishment

^a^REML did not converge, DerSimonian-Laird method was used to obtain tau

**Fig. 3:**
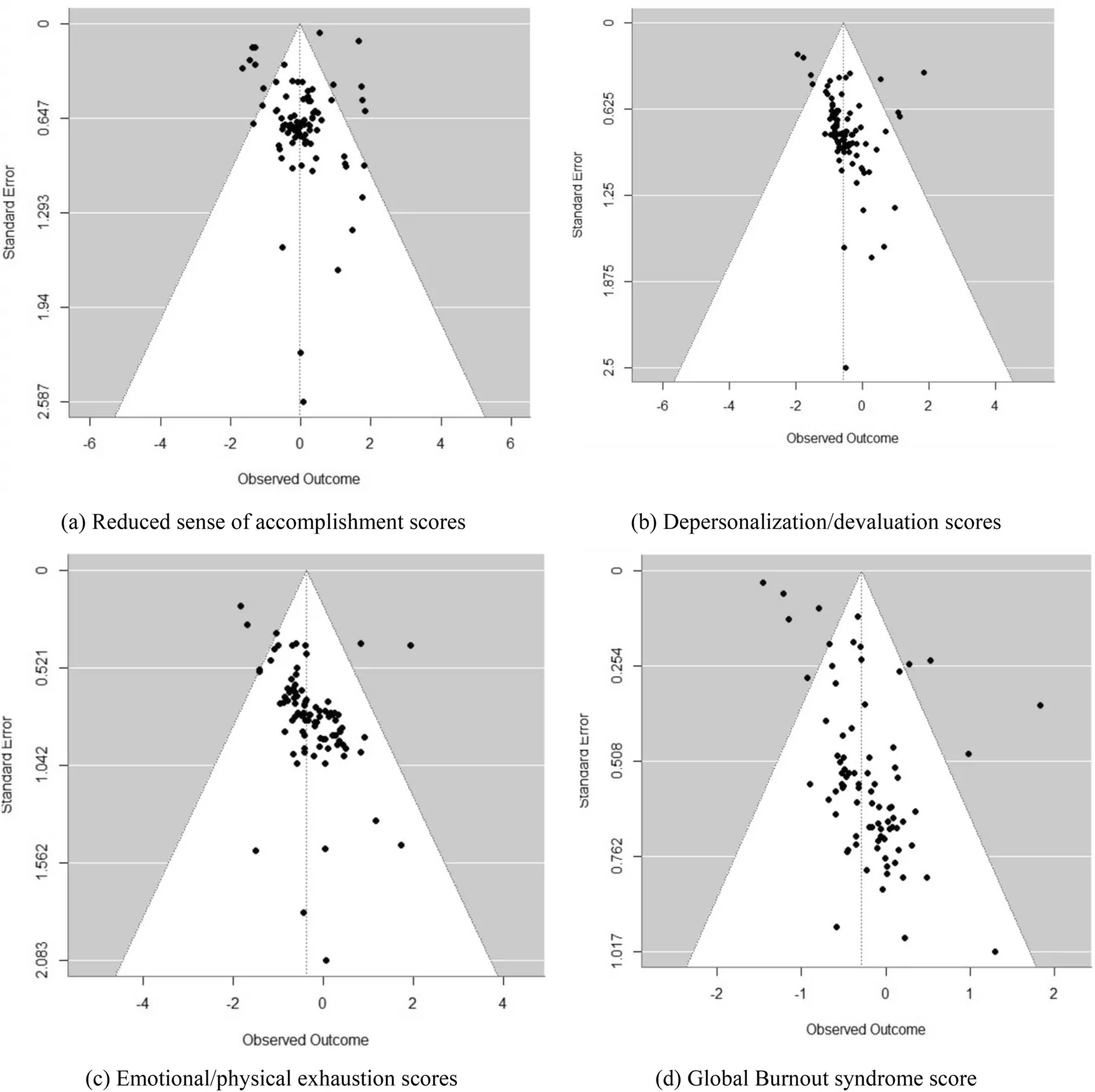
Funnel plots for each subscale and global burnout syndrome scores

Funnel plots are exhibited in Fig. 3 where outcomes (BS scores) are contrasted with the standard error of the study. Asymmetries would be a sign of publication bias.

One of the most striking results is the considerable number of studies with relatively low values of standard error but unusual extreme values of BS score. There are no apparent asymmetries in RSA scores. On the contrary, DEV, EPE and global BS scores exhibit possible asymmetry to the right (Fig. 3, panels b, c and d). In particular, the funnel plots show a certain degree of asymmetry, with smaller studies (i.e., those with higher standard errors) tending to report higher burnout scores, while larger and more precise studies cluster around lower scores. This pattern suggests the presence of small-study effects, where studies with limited sample sizes yield more extreme estimates of burnout than larger studies.

Egger tests support the analysis of publication biases and judge the significance of asymmetries, more difficult to assess in funnel plots (Table 3). The negative intercept indicates a deviation from funnel plot symmetry, and the positive coefficient for the standard error shows that effect estimates increase as study precision decreases. In other words, studies with higher standard errors—typically smaller studies—tend to report higher levels of burnout. Together, these findings provide evidence of asymmetry compatible with publication bias or other small-study effects (Table 3).

**Table 3:** Egger tests for the four burnout syndrome scores

	RSA	DEV	EPE	Global
Intercept (*p*-value)	− 0.264 (0.196)	− 0.052 (0.879)	− 0.593* (0.013)	− 0.792** (0.002)
Standard deviation (*p*-value)	0.398 (0.119)	0.142 (0.728)	0.883** (0.002)	1.526** (< 0.0001)
Adjusted R2	0.016	− 0.021	0.087	0.135

*DEV* depersonalization/devaluation, *EPE* emotional/physical exhaustion, *RSA* reduced sense of accomplishment

*p < 0.0, **p < 0.01

Table 4 exhibits the results from the multilevel meta-regression, which aids in explaining the heterogeneity found in the meta-analysis. In this case, several moderators were considered to assess their impact on BS: year of publication, sample size, type of instrument used, average age of the professionals, proportion of women, type of activity, whether the study was conducted in a developed country, and whether it considers athletes as workers.

**Table 4:** Multilevel meta-regression results

	RSA	DEV	EPE	Global
Intercept	− 90.814 (0.258)	− 1.574 (0.983)	26.140 (0.698)	124.375* (0.041)
Scale (ABQ = reference)				
IBD-R	− 0.359 (0.152)	− 0.650*** (0.004)	− 0.881*** (< 0.0001)	− 0.427 (0.147)
MBI	1.670** (0.014)	− 0.256 (0.680)	− 0.267 (0.670)	− 0.151 (0.773)
Other	− 1.604 (0.167)	− 1.288 (0.190)	− 0.182 (0.844)	0.652 (0.133)
Sample size	− 0.0001 (0.783)	0.0001 (0.892)	− 0.0001 (0.868)	0.0001 (0.948)
Type of activity (coach + referees = reference)				
Individual sports	− 0.004 (0.996)	0.159 (0.783)	− 0.164 (0.802)	− 0.87 (0.367)
Team sports	0.400 (0.558)	0.230 (0.687)	− 0.033 (0.960)	− 1.4 (0.145)
Multi-sports	0.220 (0.735)	0.474 (0.384)	0.244 (0.698)	− 0.919 (0.33)
Year of publication	0.045 (0.260)	0.001 (0.998)	− 0.013 (0.692)	− 0.061* (0.044)
Participants' average age	− 0.003 (0.908)	0.024 (0.285)	− 0.006 (0.783)	− 0.055** (0.012)
Proportion of women	− 0.129 (0.778)	− 0.244 (0.560)	− 0.017 (0.964)	− 0.337 (0.33)
= 1 if study relates to work	1.054 (0.178)	0.472 (0.543)	0.309 (0.611)	− 0.425 (0.647)
Developed country/region	0.317 (0.192)	0.176 (0.429)	0.374* (0.079)	− 0.197 (0.325)
*K*	75	74	78	56
Deviance	125.066	122.380	124.712	60.077
QE test	90.288** (0.011)	44.013 (0.950)	45.070 (0.972)	46.38 (0.335)
QM test	23.971** (0.021)	14.257 (0.285)	22.828** (0.029)	22.582** (0.032)

*p* Values are in parentheses

*ABQ* Athlete Burnout Questionnaire, *DEV* depersonalization/devaluation, *EPE* emotional/physical exhaustion, *IBD-R* Revised Burnout Inventory for Athletes, *MBI* Maslach Burnout Inventory, *RSA* reduced sense of accomplishment

*p < 0.1, **p < 0.05, ***p < 0.01

The intercept represents the average burnout score when all moderators are zero. In this case, the intercepts vary for each subscale and the overall score, but they are not significant in most cases, except for the overall measure.

In the subscales of DEV and EPE as well as for the global scale, the results from the QE test indicate the absence of residual heterogeneity. This implies that the moderators jointly explain a major part of the heterogeneity in results. There is still some residual heterogeneity in the subscale for RSA. The QM test, which checks the joint significance of the moderators, rejects the null in most meta-regressions except for DEV. That contradictory result may originate from over-adjustment.

Meta-regression shows that IBD-R produces significantly lower burnout scores for depersonalization and emotional exhaustion subscales than ABQ. On the other hand, the MBI tends to increase the score of the RSA subscale compared with the ABQ. Also, global burnout measures tend to decrease with age. Recent studies published (and probably conducted) more recently exhibit lower BS estimates.

In the meta-regression, the moderator *workrel* (indicating whether the study considers sport professionals as workers) did not show a significant association with burnout scores in any of the subscales or in the overall scale.

## Discussion

We discuss the results following the research questions initially posed as the guiding framework of the study.

*What are the different psychometric instruments used to measure burnout in professional athletes? Are there significant differences between them?*

There are various psychometric instruments available. However, the ABQ is the most widely used instrument (~ 77%) to measure burnout in athletes and other related occupations. This suggests it is broadly accepted and considered reliable within the sports research community. Nonetheless, some authors recommend adjusting the ABQ in certain items to improve its accuracy and alignment with the theoretical framework of sports burnout [39]. For instance, they propose replacing generic terms such as "negative feelings" with more precise descriptors like "lack of enthusiasm" or "cynicism," which align better with the theoretical characteristics of sports burnout. These modifications are considered to contribute to more accurate measurement and clearer interpretation of the factors comprising this important construct in sports psychology.

However, we must highlight some differences between the psychometric tools emerging in the meta-analysis. For instance, the IBD-R tends to produce lower scores in depersonalization and emotional/physical exhaustion compared with the ABQ. One possible explanation of this finding is that IBD-R focuses more on physical tiredness, while ABQ centers on emotional exhaustion. Thus, our findings indicate that workers in sports would have more mental than physical fatigue. Likewise, ABQ gives greater emphasis on capturing negative relationships between the individual and his/her colleagues or other individuals while IBD-R tries to register the internal disconnection of the athletes, coaches or referees with the activity itself. The greater score of ABQ in this dimension expresses that jobs related to sport experience higher burnout levels in terms of personal relationships relative to internal detachment.

The meta-regression also highlights that the choice of measurement instrument is a significant factor in assessing burnout in athletes and sports occupations, as some instruments produce significantly lower or higher scores in certain subscales. Therefore, the results of this study align with others [40] suggesting that while the instruments measure similar constructs, they do so in ways that may lead to differences in scores.

Additionally, < 9% of studies on athlete burnout adopt an approach that considers their participants as workers and recognizes that their sports activity occurs within the context of an employment relationship (variable "work-related"). This implies that they are subject to workplace conditions (contracts, performance expectations, etc.) within the context of a contract, which should consider aspects such as job security, working conditions, employer support, and labor rights. This disconnection between burnout and the workplace context in sports suggests a departure from the original understanding of the syndrome. This is also noted by other studies, which pointed out that original studies on burnout, which highlighted concerns about workers' occupational health, were transferred to the sports domain with approaches removing its labor aspect [34]. Although the same dimensions of burnout were recognized in athletes, the foundational understanding of the syndrome was largely overlooked in favor of more individualized approaches.

Although a low proportion of studies explicitly frame burnout in athletes as work-related, this distinction does not affect pooled prevalence estimates. This suggests that burnout prevalence is primarily driven by chronic exposure to performance demands and institutional pressures, rather than by the formal conceptual framing adopted by researchers. However, this convergence should not be interpreted as conceptual equivalence: the limited recognition of athletes as workers remains a structural limitation of the literature, restricting the integration of occupational health and labor frameworks into the interpretation of burnout in sport.

The meta-regressions show that the focus on whether burnout is a consequence of an employment relationship or is associated with other factors does not add heterogeneity to the results. Nevertheless, we agree with the proposal put forward by Akhrem and Gazdowska [41], where the authors emphasize that future research instruments should focus on establishing a more holistic framework. This framework should consider both the symptoms of burnout and the structural and contextual factors that contribute to it, thereby facilitating a more comprehensive and nuanced understanding of burnout in the sports domain. Recent literature has begun to explore this line of inquiry [42, 43].

*What is the prevalence of burnout in professional athletes? Are there potential biases in the studies reporting this data?*

Regarding the prevalence of burnout in professional athletes, the general trend in global indices points to relatively low or moderate scores, which aligns with findings from other studies [40]. The same applies when analyzing its specific dimensions.

Regarding depersonalization, it is considered less pertinent in the sports domain, as contacts do not always have the same intense interpersonal nature as in human services. Athletes may experience burnout in ways that do not necessarily involve emotional detachment from others. Instead of depersonalization, athletes may experience a devaluation of sports practice, a dimension more specific and relevant to the sports context. This devaluation refers to losing interest and meaning in sports practice, which may better represent how burnout manifests in athletes. In sports contexts, it is more appropriate to focus on the devaluation of sports practice rather than depersonalization to better capture the experience of burnout in athletes. This specific dimension, contained in instruments like the ABQ, might be a more suitable measure to reflect the detachment and loss of interest athletes may feel toward their sport, though not all instruments include it.

The average scores appear slightly higher in reduced sense of accomplishment than in the other dimensions, showing that the interventions or factors affecting depersonalization and emotional and physical exhaustion do not have a similar impact on personal accomplishment. This phenomenon could express itself in athletes receiving psychological support that helps them feel less drained and more connected with their team, but it does not change how they perceive their achievements. This suggests that improving overall burnout also requires some progress on how athletes recognize their accomplishments and personal satisfaction. However, specialized literature [41] indicates that personal accomplishment may be more challenging to address and recover due to its intrinsic and subjective nature. In contrast, depersonalization and emotional exhaustion may be more susceptible to direct and structured interventions with sports psychology professionals.

Our findings contrast with those of Madigan et al. [31], who concluded that athletes’ average burnout levels increased between 1997 and 2019. Specifically, the average levels of reduced personal accomplishment and depersonalization/devaluation have risen. The differences in the time periods covered by the referred study and ours (from 2014 to 2023) may be emerging from improvements in burnout management and greater support for athletes’ mental health available in recent years—an essential aspect for preventing all types of problems [43].

On the other hand, asymmetries observed in the funnel plot for emotional/physical exhaustion scores and global burnout scores indicate potential publication biases in these areas. The rightward asymmetry in the scores suggests that small studies (e.g., those with higher standard deviations) tend to show higher burnout scores. This bias could distort reality, creating the impression that emotional exhaustion is more prevalent than it truly is. Similarly, regarding the global burnout score, the asymmetry indicates that studies with lower global burnout scores may also be underrepresented in the published literature. Studies with more moderate or less concerning results are often underreported, leading to an overrepresentation of studies with more extreme findings.

*How do factors such as the year of publication, age, sex, type of sport, and socioeconomic context influence the levels of burnout detected in professional athletes?*

The median year of publication is 2020, with studies spanning from 2014 to 2023. This indicates a possible growing and recent interest in research on burnout in professional athletes. This increase may be related to greater awareness of mental health in sports and the need to address burnout more effectively. Additionally, the decrease in burnout scores in more recent studies suggests a potential improvement in burnout management in recent years.

Regarding age, older athletes exhibit lower prevalence of BS, indicating that burnout is gradually better managed as resilience is developed with age.

The results of the meta-regression suggest that there is no significant difference according to sex. This contrasts with previous studies indicating that women may be more susceptible to burnout and experience more severe negative consequences than men in all burnout dimensions [44, 45]. Furthermore, the average proportion of women in the studies considered in the meta-analysis is 38.5%, which highlights a still unequal representation of women and men. Future research could include a more balanced sample to obtain more representative and accurate results.

Regarding the type of sport (individual/team), no significant effects are observed in burnout scores. This result suggests the presence of a cross-instrument invariance in burnout scores, indicating that the observed pattern is not specific to the ABQ but extends to other psychometric instruments [39].

On the contrary, the socioeconomic context does influence the prevalence. According to the multilevel meta-regression, studies conducted in developed countries tend to report higher scores in the emotional exhaustion dimension. This result is remarkable, as it is often thought that, on the one hand, developed countries have more resources (economic, personal, material, etc.) and more institutional support, which can help reduce burnout. On the other hand, in developing countries, training conditions may be less favorable (lower quality facilities, less access to advanced technology, etc.), and socioeconomic conditions may emphasize the need to balance sports with other jobs or family responsibilities, producing increased stress and burnout. However, the meta-regression finds an opposite relationship. This suggests that despite having more resources and institutional support, athletes in developed countries face higher expectations and competitive pressures, contributing to higher levels of exhaustion. The higher levels of exhaustion may reflect a combination of structural and cultural factors characteristic of high-performance sport in these contexts. Athletes in wealthier countries are often embedded in highly professionalized sport systems, where competitive pressures, training intensity, and performance expectations tend to be higher. These environments may promote early specialization, rigorous workloads, and continuous monitoring, all of which can increase the risk of chronic stress and emotional exhaustion. Moreover, access to sophisticated support structures may also make it easier to detect and report symptoms of burnout, leading to more accurate or elevated scores relative to settings where psychological distress is less frequently assessed or openly discussed. Together, these dynamics may help explain why exhaustion levels appear higher among athletes from developed countries.

The finding of higher exhaustion levels in developed countries can also be theoretically interpreted through structural and organizational dynamics rather than individual-level vulnerability. Advanced economies are characterized by higher degrees of labor market intensification, performance monitoring, productivity pressures, and normative expectations of self-optimization, which systematically increase chronic psychological demands. Within such contexts, exhaustion emerges not primarily from material deprivation, but from cognitive, emotional, and temporal overload, sustained performance pressure, and the internalization of productivity norms. In this sense, developed contexts may generate structurally higher exposure to continuous performance regulation, emotional labor, role acceleration, and boundary erosion between work and non-work domains, producing elevated exhaustion despite higher material resources. This interpretation situates the observed cross-national differences not as a paradox, but as a manifestation of structurally distinct stress regimens, where burnout is driven less by deprivation and more by systemic overactivation, normative performance cultures, and institutionalized demand escalation.

We must acknowledge some limitations implicit in our analysis. Some subscales showed non-convergence under the usual method applied in meta-regression, REML, a known issue when the between-study heterogeneity is particularly small, the number of available studies is limited, or moderators suffer from severe collinearity. To assess robustness, we re-estimated τ^2^ using alternative estimators such as DerSimonian–Laird. However, this alternative is known to underestimate heterogeneity and to produce overly narrow confidence intervals, especially in small samples. Importantly, pooled estimates obtained with DerSimonian-Laird differed only marginally from those based on feasible REML, suggesting that the substantive conclusions remain stable across estimation methods. Nevertheless, results for these specific subscales should be interpreted with caution given the technical limitations associated with both REML non-convergence and the alternative estimator's known biases.

Another limitation of the study is the potential existence of publication biases that may have influenced the results, overestimating burnout prevalence. Additionally, most studies were conducted in developed countries, which may not fully reveal the situation in developing countries. Finally, it is important to highlight that most of the included studies are cross-sectional, which limits the ability to assess changes in burnout over time.

## Conclusion

This study has allowed for the evaluation of the prevalence and contributing factors of burnout syndrome in professional athletes and sport-related occupations through a meta-analysis. The main findings indicate that, firstly, the ABQ is the most commonly used instrument to measure burnout in athletes, followed by the IBD-R and the MBI. Significant differences exist between these instruments, particularly in how they measure the subscales. In particular, MBI may underestimate the BS related to personal achievement.

Secondly, the general trend in global burnout indices in professional athletes shows relatively low to moderate scores. In addition, there is some evidence of a negative trend in global burnout figures in recent years.

Third, the factors that significantly influence burnout are age and socioeconomic context. Older athletes tend to experience less burnout, implying greater resilience with age. Studies conducted in developed countries tend to report higher exhaustion scores, possibly due to greater expectations and competitive pressures. Factors such as sex or type of sport do not significantly influence burnout scores. Nevertheless, the set of moderators selected adequately captures the overall variability in BS scores, especially in emotional exhaustion and global burnout scales, where residual heterogeneity vanishes.

Fourth, potential publication biases were detected, especially in the dimensions of exhaustion and global burnout scores, which could lead to an overestimation of the prevalence and severity of burnout.

### Practical Implications

It is crucial to improve the burnout measurement tools to better reflect athletes' experiences. Adjustments to the ABQ and consideration of contextual and structural factors could contribute to a more accurate assessment. Additionally, the occupational dimension of professional sports should emerge as a central element in understanding burnout. Recognizing the athlete as a worker involves not only addressing their competitive demands but also ensuring working conditions that preserve their overall well-being, moving toward approaches that integrate human and professional dimensions.

The results suggest the need to develop personalized interventions that specifically address emotional and physical exhaustion, as well as the devaluation of sports practice. These interventions should consider the athletes’ age (paying special attention to younger athletes) and their socioeconomic context.

### Future Proposals

We suggest conducting longitudinal studies that allow for the assessment of burnout evolution in athletes over time and the effectiveness of professional interventions. It would also be pertinent to include more studies from developing countries and a balanced representation of women and men in order to obtain a more global and representative view of burnout in sports-related jobs.

## Supplementary Information

Below is the link to the electronic supplementary material.

Supplementary file1 (CSV 36 KB)

## Abbreviations

ABQ: Athlete Burnout Questionnaire
BIR: Burnout Inventory for Referees
BS: Burnout Syndrome
BVS: Biblioteca Virtual en Salud (Virtual Library on Health)
CBI: Copenhagen Burnout Inventory
CBQ: Coach Burnout Questionnaire
DEV: Depersonalization
EPE: Emotional exhaustion
IBD: Burnout Inventory for Athletes
IBD-R: Revised Burnout Inventory for Athletes
ICD: International Classification of Diseases
MBI: Maslach Burnout Inventory
PRISMA: Preferred Reporting Items for Systematic reviews and Meta-Analyses
REML: Restricted maximum likelihood estimator
RSA: Reduced sense of accomplishment
SMBM: Shirom–Melamed Burnout Measure
SpBI-DC: Sport Burnout Inventory Dual Career
WHO: World Health Organization

## Declarations

### Availability of data and material

The data supporting the findings of this study are available from the corresponding author upon request.
